# ClinLabGeneticist: a tool for clinical management of genetic variants from whole exome sequencing in clinical genetic laboratories

**DOI:** 10.1186/s13073-015-0207-6

**Published:** 2015-07-29

**Authors:** Jinlian Wang, Jun Liao, Jinglan Zhang, Wei-Yi Cheng, Jörg Hakenberg, Meng Ma, Bryn D. Webb, Rajasekar Ramasamudram-chakravarthi, Lisa Karger, Lakshmi Mehta, Ruth Kornreich, George A. Diaz, Shuyu Li, Lisa Edelmann, Rong Chen

**Affiliations:** Department of Genetics and Genomic Sciences, Icahn Institute for Genomics and Multiscale Biology, Icahn School of Medicine at Mount Sinai, New York, NY USA

## Abstract

Routine clinical application of whole exome sequencing remains challenging due to difficulties in variant interpretation, large dataset management, and workflow integration. We describe a tool named ClinLabGeneticist to implement a workflow in clinical laboratories for management of variant assessment in genetic testing and disease diagnosis. We established an extensive variant annotation data source for the identification of pathogenic variants. A dashboard was deployed to aid a multi-step, hierarchical review process leading to final clinical decisions on genetic variant assessment. In addition, a central database was built to archive all of the genetic testing data, notes, and comments throughout the review process, variant validation data by Sanger sequencing as well as the final clinical reports for future reference. The entire workflow including data entry, distribution of work assignments, variant evaluation and review, selection of variants for validation, report generation, and communications between various personnel is integrated into a single data management platform. Three case studies are presented to illustrate the utility of ClinLabGeneticist. ClinLabGeneticist is freely available to academia at http://rongchenlab.org/software/clinlabgeneticist.

**Electronic supplementary material:**

The online version of this article (doi:10.1186/s13073-015-0207-6) contains supplementary material, which is available to authorized users.

## Background

Molecular genetic testing is playing an increasingly important role in medicine. Due in large part to the breakthrough of genome and exome sequencing technologies, the scope of clinical genetic testing has been expanded from its traditional niche in rare Mendelian disorders to a broad application in complex disease and personalized medicine [1, 2]. Currently, clinical genetic testing is utilized for a variety of purposes including follow-up to newborn screening for the identification of genetic disease that may affect a child’s long-term health or survival, carrier screening for inherited recessive and X-linked diseases, diagnostic testing for symptomatic individuals, predictive testing of asymptomatic individuals for late-onset and complex diseases, pharmacogenetic testing for drug responses with respect to efficacy or adverse effects, and testing of tumor biopsies to determine somatic alterations for cancer classification, prognosis, and development of personalized treatment options [1].

There are a number of challenges in applying whole exome sequencing (WES) in clinical genetic testing. Although most clinical genetic testing laboratories follow the guidelines from national and international agencies such as American College of Medical Genetics (ACMG), College of American Pathologists (CAP), and Clinical and Laboratory Standard Institute (CLSI), tools are lacking to bridge these guidelines and clinical practice. In addition, there are a large number of variants of uncertain significance (VUS). As basic research accelerates with improved technology and more discoveries are made toward the genetic basis of human diseases, it is critical to incorporate the most updated and comprehensive genetic variant findings into clinical genetic testing. In addition, previously completed testing reports may need to be updated when new information becomes available on the function and pathogenicity of the identified variants. Genetic testing in clinical laboratories involves a complicated process, which requires efficient data management and process management with seamless coordination and communication between various personnel. A final report for each patient is expected to precisely summarize the current knowledge surrounding the variants and their clinical implication with supporting evidence, and the report should be generated in a comprehensive fashion. Currently, although many clinical laboratories use commercial tools to annotate variants in a semi-automated fashion, variant assessment still involves manual inspection of different online databases and copy-paste of relevant content into the report. Many laboratories still use Excel files to manage variant datasets. As the volume of WES testing increases, this practice is inefficient, error-prone, and unscalable. Therefore, in order to facilitate the implementation of WES-based genetic testing, an integrative tool is essential to provide a comprehensive data source for variant assessment, and to automate, therefore, enhance the efficiency of the process and reduce potential errors that may arise in handling large datasets.

There are several commercial tools currently available for variant analysis and interpretation. For example, Ingenuity Variant Analysis tool by QIAGEN [3], Geneticist Assistant by SoftGenetics [4], VarSeq by Golden Helix [5], VarSim by Bina Technology [6], ANNOVAR Tute annotation from Tute Genomics [7], and The Exchange by NextCode [8] allow users to import VCF files after initial processing of sequencing data, followed by variant filtering based on data-related parameters such as supporting sequence reads or allele frequency. Subsequently, users can further explore the data to examine if the variants are present in various databases such as dbSNP [9] for known polymorphisms and their population allele frequencies, or in disease related variant databases such as ClinVar [10], OMIM [11], HGMD [12], and COSMIC [13]. Potential functional consequences of the variants can also be assessed using methods such as SIFT [14], PolyPhen [15], and SeqHBase [16, 17]. Most of these tools also provide a genomic viewer for visualization of variants and sequence alignment. Some of the tools, such as NextCode’s The Exchange, even allow user-controlled data sharing. However, most of these tools are designed primarily for research purposes and others do not meet all the needs of a clinical laboratory. GeneInsight Suite is a tool developed to support use of DNA-based genetic testing by clinical laboratories and health providers [18]. However, it was primarily designed for clinical variant data storage, variant classification, and report generation.

Previously, we reported a comprehensive validation study for WES implementation in the Genetic Testing Laboratory at Mount Sinai [19]. We tested parameters that measure the reproducibility of the sequencing platform as well as the informatics pipelines. Our evaluation focused on SNV and small indel detection for a single workflow across multiple technical replicates. This study validated the analytic performance of WES according to the recommended guidelines [20], and established the foundation of WES-based genetic testing at Mount Sinai. In this report, we describe a tool named ClinLabGeneticist specifically designed to enable and facilitate WES testing in a clinical genetic laboratory setting. We have established a comprehensive data repository for variant annotation including all of the publicly available databases, to our knowledge, for non-disease or disease-related variants. This application provides a platform to automate data management and process management for the highly complex genetic testing workflow, significantly improving the efficiency of clinical WES testing.

## Implementation

### WES and ClinLabGeneticist workflow

**Fig. 1:**
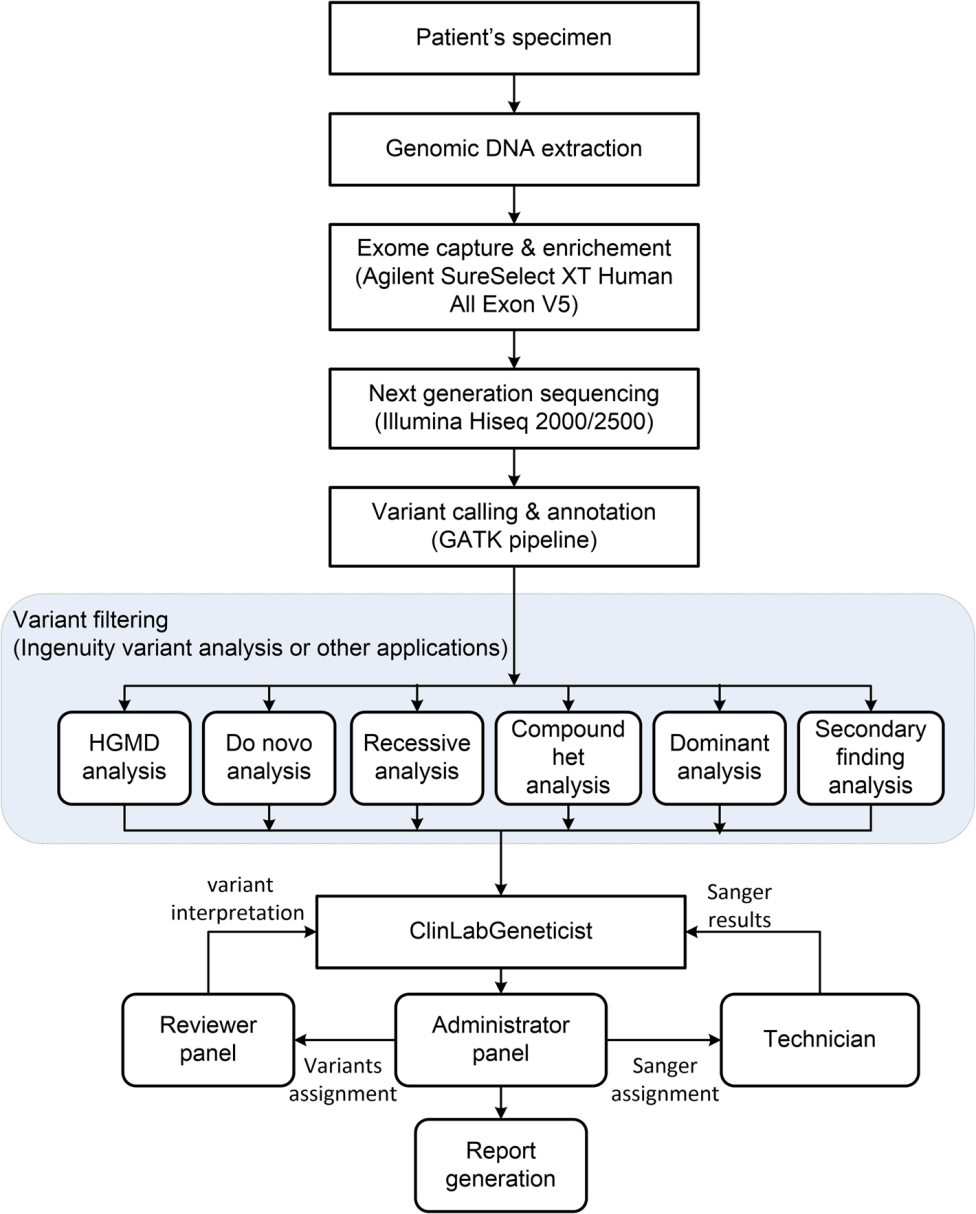
WES workflow at Mount Sinai Genetic Testing Laboratory

The overall WES workflow at Mount Sinai Genetic Testing Laboratory is illustrated in Fig. 1. For whole exome sequencing, genomic DNA was extracted from the peripheral blood samples of patients and exonic regions were enriched by Agilent SureSelect XT Human All Exon V5 capture library. Massively parallel sequencing was performed on an Illumina HiSeq2000/2500 with a 100 bp paired-end protocol. The genome analysis pipeline or GAP, which is based on the 1000 Genomes data analysis pipeline and is composed from the widely-used open source software projects including bwa, Picard, GATK, snpEff, BEDTools, PLINK/SEQ, and custom-developed software was used for variant calling and annotation [19]. VCF files generated by GAP were then uploaded into Ingenuity Variant Analysis tool (QIAGEN) for further variant filtering. Based on patients’ clinical and family history, multiple analyses were performed in Ingenuity including HGMD analysis (for searching disease-causing mutations or DM reported in HGMD database), *de novo* analysis (for searching *de novo* variants), dominant analysis (for dominant inheritance pattern), recessive analysis, compound heterozygous analysis (both for recessive inheritance and X-linked patterns), and secondary finding analysis (based on ACMG incidental finding gene list) [21]. Variant lists generated by these Ingenuity analyses were then used as input for the ClinLabGeneticist software. Users can also upload input files directly into ClinLabGeneticist after variant filtering using tools such as Cartagenia Bench Suite [22] or the Clinical Sequence Analyzer (CSA) from NextCode [23]. The input file format (Additional file 1: Table S1) should include the following columns: chromosome number, chromosome coordinate, reference allele, sample allele, gene symbol, transcript ID, nucleotide alteration, amino acid alteration, SIFT functional prediction, PolyPhen-2 functional prediction, conservation phyloP p-value, dbSNP ID, and 1000 genome allele frequency. ClinLabGeneticist supports analysis of variants generated using various sequencing platforms including Ion Torrent, Agilent, Nimblegen, and others.

**Fig. 2:**
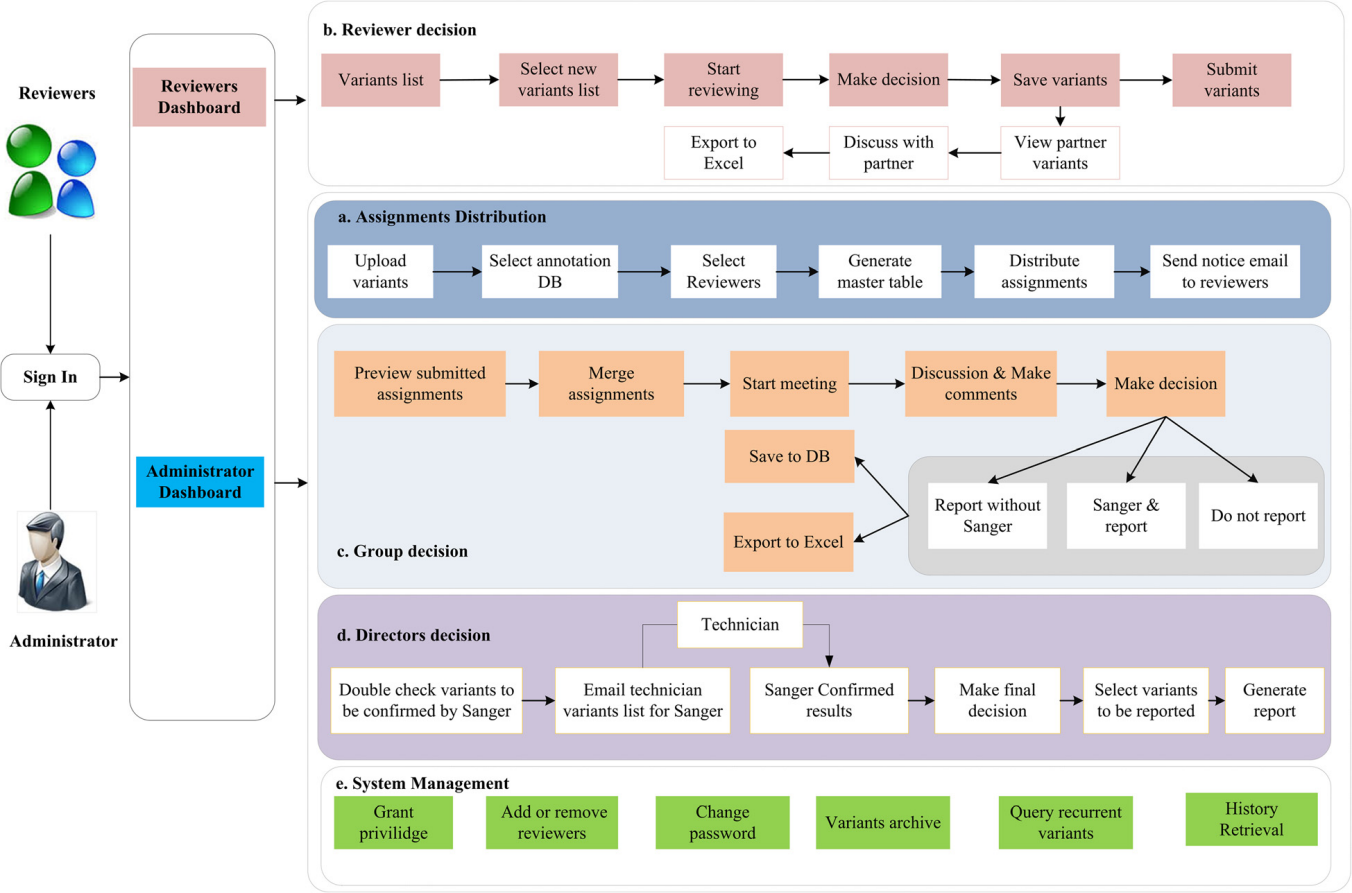
Architecture and functionalities of ClinLabGeneticist. **a** Administrator annotates and distributes variants to reviewers. **b** Reviewers review variants and make a group decision. **c** Lab director confirms variants and generates report. **d** Administrator manages reviewers, archives variants, queries recurrent variants, and retrieves history. **e** System management by system administrators

The architecture and functionalities of ClinLabGeneticist are depicted in Fig. 2. Two dashboards were designed for the administrators and the reviewers. The dashboard for the administrators enables them to accomplish the following responsibilities: (1) Upload variant data derived from a patient sample; generate a master table with variant annotations automatically retrieved from our annotation repository; select relevant annotation databases for each variant; distribute variants to different groups of reviewers; and notify reviewers of their tasks and deadlines. Each variant can be assigned to at least two reviewers for independent review. (2) Examine the results submitted by the reviewers, merge results, and highlight discordant interpretations on the same variant by different reviewers. (3) Set up reviewer group meetings for discussion, resolve discrepancies in variant interpretations, select variants for validation by Sanger sequencing, and trigger the validation process. (4) Push results to the laboratory director for final decisions on what variants to report and their interpretations, and generate variant tables for final reports.

The reviewers’ dashboard is designed to allow reviewers to review the assigned variants, provide variant analysis results and interpretations through the dashboard, and discuss with other reviewer assigned on the same variant. The system is designed to auto-save reviewers’ variant annotation every 30 s. The IGV viewer is integrated into ClinLabGeneticist to display sequence alignment for visual inspection of variants. Hyperlinks are set up for variant annotations to their corresponding external databases (for example, dbSNP, OMIM, ClinVar, and so on) upon which the annotation is based. In addition, the chromosome location of the variant is linked to the UCSC browser, gene symbol is linked to the GeneCards website for more detailed gene description, and each gene is linked to NCBI PubMed for relevant literature. Integration of these links and the IGV viewer provides tremendous convenience for the reviewers so they can perform all required tasks within the same software system without having to manually launch different tools separately.

The system is managed by a system administrator whose responsibilities include granting privileges, adding or removing reviewers, and managing variant archives.

### Variant annotation resources in ClinLabGeneticist

We developed a comprehensive variant annotation repository. The included databases, datasets, and annotation features are listed in Table 1 and Additional file 1: Table S2. They comprise publicly available databases for non-disease (for example, dbSNP, 1000 genome, UK10K, ESP6500 from NHLBI’s exon sequencing project, the Wellderly project by Scripps Insititute, and ExAC data from Exome Aggregation Consortium) or disease-related (for example, HGMD, ClinVar, OMIM, and UK10K disease) variants. In addition, data sources that are not yet available to public are incorporated, such as genotyping data from Mount Sinai Biobank, a biobank established in 2007 in New York City with ethnically diverse participants [24], and in-house curated disease variant database VarDi [25] based on manual curation and literature mining. We also added datasets for functional consequences of the variants such as dbNSFP and pre-computed results of currently known genetic variants using tools such as SIFT [14], PolyPhen [15], ANNOVAR [7], SnpEff [26], and MutationAssessor [27].

**Table 1:** Variation annotation resources in ClinLabGeneticist

Variant database	Description	Reference
dbSNP	NCBI genetic variant database	[9]
1000 Genome	1000 genome sequencing project	[35]
ESP6500	Exome sequencing project by NHLBI	[36]
UK10K control	WGS cohorts of 4,000 people in UK	[37]
Scripps Wellderly	Sequencing of 2,000 healthy elderly volunteers	[38]
ExAC	Exome aggregation consortium	[39]
dbNSFP	Functional prediction and annotation of non-synonymous SNVs	[40]
HGMD	Human gene mutation database	[12]
ClinVar	Relationship between variants and human disease phenotype	[10]
OMIM	Online Mendelian Inheritance in Man	[11]
UK10K disease	WES of 6,000 patients with neurodevelopment, obesity, and rare diseases in UK	[37, 41]
GERA	Genotyping data of 78,000 individuals with common age-related diseases	[42–44]
Mount Sinai Biobank	Genotyping data from Biobank at Mount Sinai	[24, 25]
VarDi	In-house disease variants database	[25]

### Software implementation

ClinLabGeneticist is built on the Windows platform (Window 7 and 8). Conventional client/server architectures were utilized to support concurrent and multi-users. Specifically, the machine with Windows operating system for each user is the client, and the machine with the backend MySql database and performs data query, processing, and management is the server. The server is deployed in Linux. Major functions of the administrator and the reviewers such as assignment distribution, variant annotation, assignment combination, group meeting are implemented on the Windows. All of the annotated and reviewed variants by either administrator or reviewers are saved in the database on the server. The client interface is implemented by Visual Basic, HTML, and PHP.

We recommend the following hardware specifications to run the software on the client side.

- Processor - Intel ® core™ i5-3470 @ 3.20 GHz (or equivalent AMD)
- RAM - 4 GB (or higher)
- Hard drive - 120 GB 5,400 RPM hard drive
- Wireless (for laptops) - 802.11 g/n (WPA2 support required)
- Operating system - Windows 7 or 8

Currently our backend MySql database is deployed on Mount Sinai high performance computer system which consist of 120 Dell C6145, two blade chassis nodes, 7,680 Advanced Micro Devices (AMD) 2.3 GHz Interlagos cores (64/node) and 64 compute cores in four sockets, and 256 Gigabytes (GB)s of memory per node. A detailed instruction on software installation and setup of internal server and backend databases is available as a power point file on software’s homepage [28].

### Patient consent and study approval

Informed consent for clinical exome sequencing was obtained from all patients and/or their guardians. Patients assented to have their data used anonymously for research in all cases as per New York State Department of Health requirements for informed consent.

## Results and discussions

### A comprehensive genetic variant data repository

Our variant data repository included more than 400,000 variants at approximately 360,000 variant sites from more than 10 databases (Table 1). The total number of samples with whole genome or exome sequencing data from these databases is approximately 82,000, with an additional 90,000 genotyped individuals.

### Automation of clinical genetic testing process using ClinLabGeneticist

**Fig. 3:**
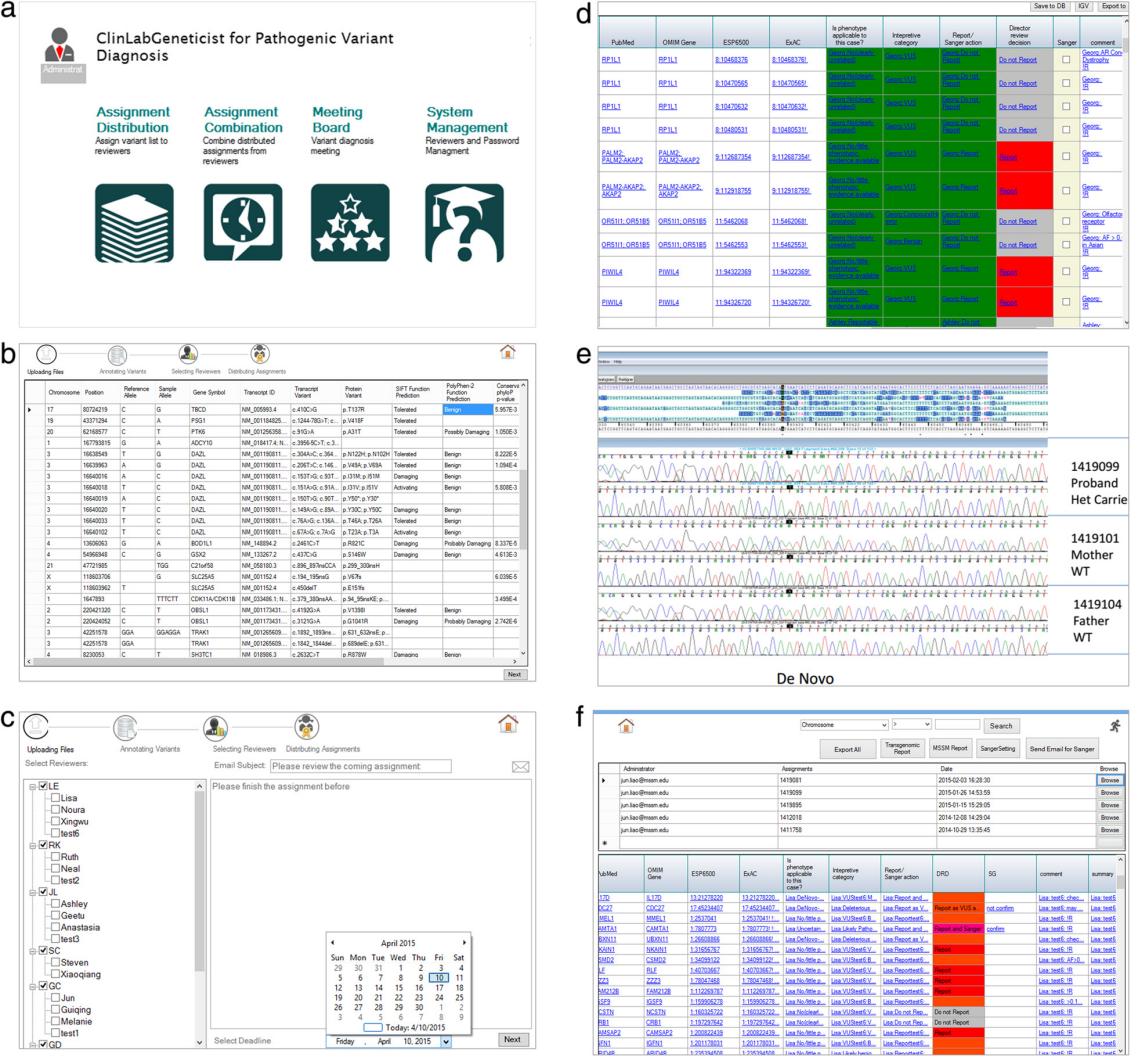
Screen shots of the administrators’ dashboard. (**a**) Dashboard, (**b**) functionalities controlled by the dashboard such as data upload, (**c**) distribute work assignments, (**d**) merge data table, (**e**) validation of variants by sanger sequencing, and (**f**) selection of variants to generate final reports

A key feature of ClinLabGeneticist is the implementation of dashboards to automate the entire workflow. Figure 3 shows selected screenshots of the administrators’ dashboard and some of the functionalities controlled by the dashboard. The dashboard (Fig. 3a) allows the administrators to upload the data (Fig. 3b), distribute the assignments with the defined timeline (Fig. 3c), highlight discordant variant evaluation results by individual reviewers (Fig. 3d), record decisions on variant interpretations and decisions on downstream validation by Sanger sequencing (Fig. 3e), and finally generate a tables of variants for the clinical report (Fig. 3f). Under hardware specification described in the software implementation section, it takes less than 10 min for an administrator to upload and annotate one variant file from WES. Annotation databases (Table 1) are not downloaded and stored on local servers. Instead, a link to the original database repository is provided so the administrator will always retrieve the latest annotations from each database.

**Fig. 4:**
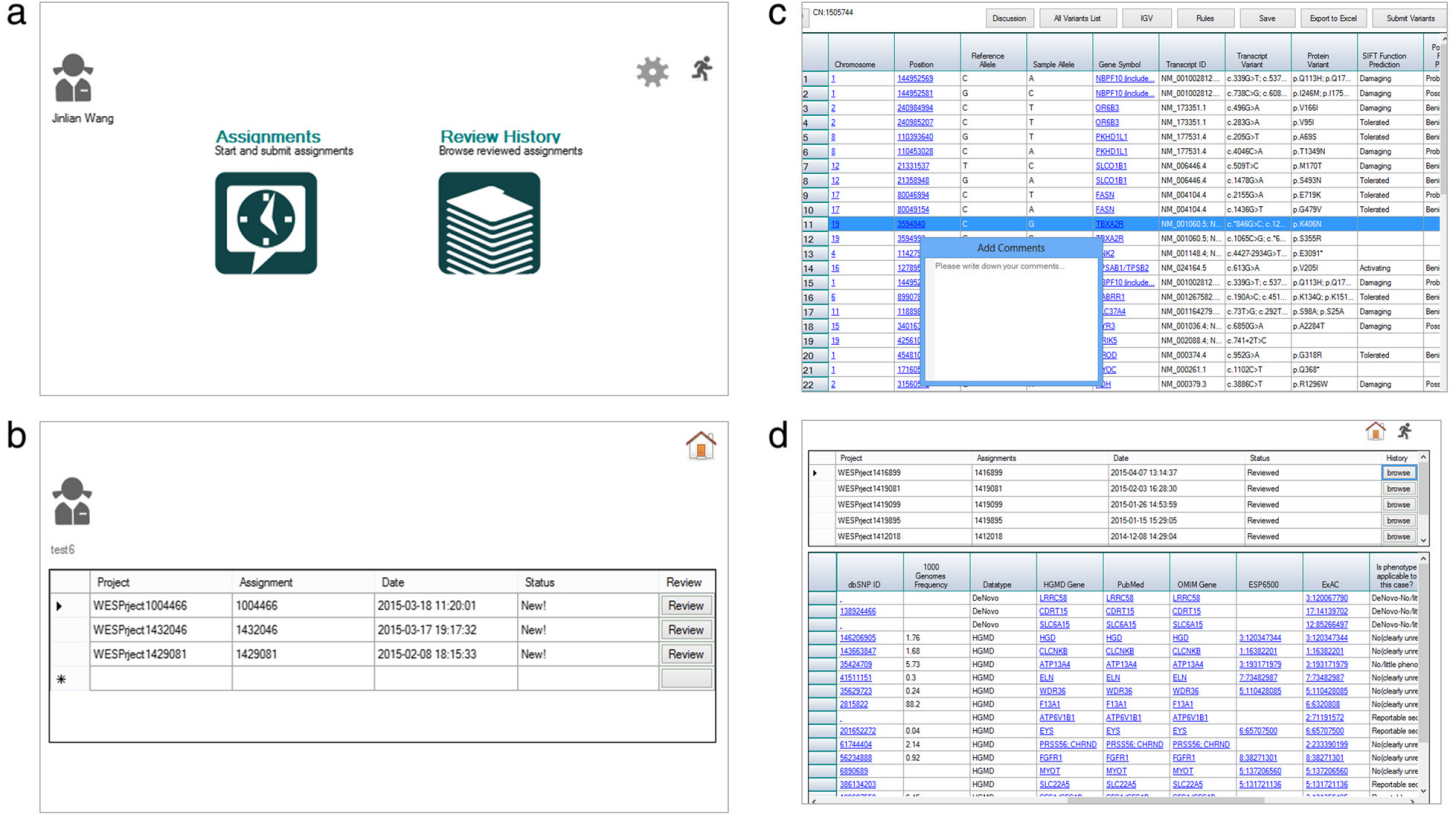
Screen shots of the reviewers’ dashboard. (**a**) Dashboard, (**b**) functionalities controlled by the dashboard such as display assigned variant lists, (**c**) review variants, and (**d**) access historic assignments and results

Reviewers’ dashboard and some of its functionalities are illustrated in Fig. 4. The dashboard (Fig. 4a) allows each reviewer to view a list of variants assigned by the administrator using the annotation databases selected by the administrator (Fig. 4b), and enables reviewers to examine relevant variant annotation data sources and references with external links in order to assess variant pathogenicity and disease association (Fig. 4c). Upon completion of evaluation, for each variant, the reviewers make a call at the gene level regarding how the phenotype of the patient relates to the disease associated with this gene (Table 2a). This is followed by a subsequent call at the variant level regarding variant pathogenic categories (Table 2b). Variant annotations from different sources may play different roles in variant assessment depending on circumstances. For example, ClinVar, HGMD and OMIM annotations are critical to determine variant pathogenicity. Variant allele frequencies in 1000 genome and ExAC are more important parameters when variants are called benign. Based on these two calls, the ClinLabGeneticist will take the following actions for the variant based on an internally developed logic (Table 3): report and proceed to validation by Sanger sequencing, report without Sanger sequencing, or do not report. The reviewers’ dashboard also allows each reviewer to browse historical assignments and review results stored in the database (Fig. 4d).

**Table 2:** Criteria for assessment of disease association at gene (a) and at variant (b) level

a. Is phenotype applicable to this case (at gene level)		
Option	Where to look	When to choose
Yes	OMIM, HGMD, PubMed	Disease clinical features match patient’s phenotype
Uncertain/possibly	OMIM, HGMD, PubMed	Disease clinical features partially overlap with patient’s phenotype
No (clearly unrelated)	OMIM, HGMD, PubMed	No overlapping phenotype, totally different disease
No/little phenotypic evidence available	OMIM, HGMD, PubMed	Phenotypic evidence was only found in few low-quality papers, or only from association studies, or only somatic mutations were reported
*de novo* - No/little phenotypic evidence (chose for variants from *de novo* filter only)	OMIM, HGMD, PubMed	Same as ‘No/little phenotypic evidence available’, but only for *de novo* variants
Reportable secondary finding	OMIM, HGMD, PubMed	Depends on patient’s requirement, mostly for genes associated with actionable diseases. Not limit to genes in ACMG guideline. If the patient does NOT want secondary findings, do NOT choose this option
b. Interpretive category (at variant level except deleterious VUS)		
Option	Where to look	When to choose
Benign	1000 Genomes, EVS, ExAC	Allele frequency >1 % for recessive or X-linked patterns. And for X-linked pattern, at least several hemizygous males should be reported in the database. Or allele frequency >0.1 % for dominant or *de novo* patterns
Likely benign	UCSC genome browser	Deletion/insertion of 1–2 aa in a repeat region composed of at least 8 aa repeats
Intronic-likely benign	UCSC genome browser, HGMD, ClinVar	The nomenclature for all transcripts indicates that the change is intronic, but not in canonical splice sites (−1, −2, +1, or +2), except variants reported in HGMD or ClinVar as pathogenic/likely pathogenic
VUS		Variant which does not fit other categories
Deleterious VUS (only chose for genes with no/little phenotype evidence)	UCSC genome browser, ACMG guideline	Variant assumed to disrupt gene function (nonsense, frameshift, canonical splice sites, and so on), but in a gene with no/little phenotype evidence available
Likely pathogenic	UCSC genome browser, ACMG guideline	Has not been reported before, but is assumed to disrupt gene function (nonsense, frameshift, canonical splice sites, and so on). Or variant which meets ACMG guideline
Pathogenic	HGMD, ClinVar, OMIM	Well-established disease-causing mutation by previous reports
Mapping error	UCSC genome browser, IGV, Ingenuity	Variant in segmental duplication or repeat region, and mapping quality/coverage is low. Generally you can see many variant calls in the same region. Also pay attention to complex variants such as large deletions/insertions and indels, please check IGV because nomenclature could be wrong
CompoundHet error	Ingenuity	Only 1 non-benign variant found in a gene. Only use for variants that pass through the Compound Het filter

**Table 3:** Logic for variant reporting and validation by Sanger sequencing

Interpretive category	Phenotype applicable					
	Yes	No (clearly unrelated)	Reportable secondary finding	Uncertain/possibly	No/little phenotypic evidence available	*de novo* - No/little phenotypic evidence (chose for variants from *de novo* filter only)
Benign	Do not report	Do not report	Do not report	Do not report	Do not report	Do not report
Likely benign	Report & Sanger	Do not report	Do not report	Report & Sanger	Report	Report & Sanger
Intronic-likely benign	Report & Sanger	Do not report	Do not report	Report & Sanger	Do not report	Do not report
VUS	Report & Sanger	Do not report	Do not report	Report & Sanger	Report	Report & Sanger
Deleterious VUS (only chose for genes with no/little phenotype evidence)	Error - please change category	Error - please change category	Error - please change category	Error - please change category	Report as VUS	Report as VUS & Sanger
Likely pathogenic	Report & Sanger	Need discussion	Report as secondary & Sanger	Report & Sanger	Error - please change to Deleterious VUS	Error - please change to Deleterious VUS
Pathogenic	Report & Sanger	Need discussion	Report as secondary & Sanger	Report & Sanger	Error - please change to Deleterious VUS	Error - please change to Deleterious VUS
Mapping Error	Investigate further via Sanger	Do not report	Do not report	Need discussion	Do not report	Do not report
CompoundHet error	Investigate further via Sanger	Do not report	Need discussion	Need discussion	Do not report	Error - not compound het

After ClinLabGeneticist was launched, we have evaluated more than 17,000 variants in 245 genes associated with 53 diseases. For most variants that lack clear evidence as pathogenic variants, it takes only 1–2 min to complete the review process using ClinLabGeneticist. For those variants with substantial annotation and literature reports, the maximal time to complete the review process is approximately 15 min because all relevant information is displayed by ClinLabGeneticist with external links and the IGV viewer automatically launched, allowing the reviewers to navigate the information with ease. Before ClinLabGeneticist was developed, variant Excel files were generated and distributed to each of the first reviewers for their variant assessment. The clinical laboratory directors or second reviewers will have to consolidate and compare first reviewers’ assessment to prioritize variants for follow-up studies such as Sanger validation and categorization for final reporting. This manual workflow was transformed by the implementation of ClinLabGeneticist to become automated and therefore reduced the administrative effort by at least 50 %. In addition, all of the variants are annotated in ClinLabGeneticist in a fully customized manner which is essential to improve overall work efficiency and accuracy in a clinical lab. The reviewers will not need to search for annotations in different public or private databases manually. More importantly, most of the public or private variant databases are not designed for clinical use and they have to be curated and customized for clinical implementation, which can be accomplished in ClinLabGeneticist. In the following section, we present three case studies to further illustrate the utility of ClinLabGeneticist. *De novo*, recessive, compound heterozygous, and secondary variants in each case were analyzed. Described in Additional file 1: Table S3 are the number of variants at each step of the process, for example, concordant and discordant calls by different reviewers, decisions on variant report and Sanger sequencing validation, and variant reporting in various categories (primary, supplementary, and secondary finding). Detailed variant list for each of the three cases are provided in Additional file 1: Table S4–S6, respectively.

### Case study 1

Patient 1 was diagnosed with congenital erythropoietic porphyria (CEP) at the age of 5 months by biochemical testing and the diagnosis was later confirmed by DNA analysis showing homozygosity for the *UROS* C73R mutation, which is known to cause a severe phenotype. The patient had a bone marrow transplantation at 2 years of age due to transfusion-dependent hemolytic anemia and severe cutaneous involvement associated with CEP. However, the patient also had several other features that were inconsistent with the diagnosis of CEP, including developmental delay, congenital glaucoma, complicated retinal and ocular problems, and facial dysmorphisms. Due to the many unexplained anomalies, the patient was evaluated by a clinical geneticist in 2012. Array CGH was normal and molecular testing for Stickler syndrome revealed a heterozygous variant of uncertain significance in the *COL11A1* gene. However, these tests were performed on peripheral blood likely reflective of the bone marrow donor’s results given the complete engraftment from past transplantation.

The patient was evaluated at Mount Sinai and specimens were submitted to the Mount Sinai Genetic Testing Laboratory in February 2014 for exome sequencing on fibroblasts derived from the patient’s skin biopsy and blood samples from both parents. The sequence data were analyzed as a trio, and variants analysis was performed using ClinLabGeneticist software based on the following inheritance patterns: *de novo*, autosomal recessive. ClinLabGeneticist was used in this study to evaluate seven compound heterozygous, 22 recessive, four *de novo*, and 15 secondary variants and generate a clinical report. From the sequencing data, a homozygous pathogenic mutation, c.217T>C was identified in exon 4 of the *UROS* gene resulting in an amino acid change p.C73R. Mutations in *UROS* cause autosomal recessive congenital erythropoietic porphyria (MIM: 263700, [29]). This variant has been reported as the most frequent mutation found in CEP (CM900225 in HGMD database, RCV000003948.2 in ClinVar database, rs121908012 in dbSNP database). Sanger sequencing of DNA from the trio confirmed that the mutation was homozygous in the patient and that each of the parents was a heterozygous carrier for this variant. Therefore, the homozygous state of this variant was interpreted as a pathogenic.

Two other variants were also reported from the study. A *de novo* heterozygous variant of uncertain significance, c.2855G>T was identified in the last exon of the *INPP4A* gene resulting in an amino acid change p.R952L. *INPP4A* has not been described as a disease-related gene with substantial evidence and there is limited information in the literature regarding its function. It has been suggested that *INPP4A* plays a role in brain development as targeted disruption of the *Inpp4a* gene in mice leads to neurodegeneration in the striatum, the input nucleus of the basal ganglia that has a central role in motor and cognitive behaviors [30]. The c.2855G>T variant in *INPP4A* is predicted to be damaging by SIFT and probably damaging by PolyPhen-2. Sanger sequencing of DNA extracted from the patient and both parents confirmed that the variant occurred *de novo*. A second *de novo* heterozygous variant of uncertain significance, c.985G>A was identified in exon 11 of the *RANBP3* gene resulting in an amino acid change p.E329K. *RANBP3* has not been described as a disease-related gene with substantial evidence and there is limited information in the literature regarding its function. The variant is predicted to be damaging by SIFT and possibly damaging by PolyPhen-2. Sanger sequencing of DNA extracted from the patient and both parents confirmed that the variant occurred *de novo*. This variant was also interpreted to be of uncertain significance.

In addition to the above three variants, seven compound heterozygous variants were also reported in a supplementary table. For three of these seven variants, the initial review by two independent reviewers resulted in discrepant calls. In two cases, one reviewer called the variant ‘VUS’ while the other reviewer assigned the variant into the ‘mapping error’ category. In the third case, one reviewer called the variant ‘likely pathogenic’ and the other reviewer called the same variant ‘VUS’. Upon further examination and discussion in the group meeting, it was determined that all three variants should be called ‘VUS’ and should be reported.

In summary, exome sequencing-based genetic testing confirmed the homozygous pathogenic mutation p.C73R despite reported complete engraftment of donor bone marrow which should have precluded a positive result. No variants were identified that explained the patient’s other abnormalities though reanalysis could lead to reassignment of variant categories based on new data in the future.

### Case study 2

Patient 2 had significant developmental delay and some dysmorphic features. Previous chromosome and Array CGH analysis had not revealed any abnormalities. DNA was also tested by a targeted gene panel for autism in the Mount Sinai Medical Genetics Testing Laboratory, but no pathogenic mutation was detected. Additional metabolic screening test results were negative.

In light of the negative metabolic and genetic testing workup, whole exome sequencing was performed on DNA extracted from the patient and the parents. The sequence data were analyzed as a trio, and variants analysis was performed using ClinLabGeneticist software based on the following inheritance patterns: *de novo*, autosomal recessive. A *de novo* variant of uncertain significance was identified in exon 31 of the *PPFIA2* gene, NM_001220473.2:c.133A>G, p.Val1241Ile (hg19 Chr12:81653434). *PPFIA2* has not been described as a disease-related gene with substantial evidence. This variant has not been reported in any public population variant database and is predicted to be a ‘tolerated’ change by SIFT *in silico* analysis. Sanger sequencing of DNA extracted from the patient and both parents confirmed that the variant occurred *de novo*. In addition, this variant was not detected in the patient’s unaffected sibling.

### Case study 3

Patient 3 is a 7-year-old boy with developmental delay. He had some autistic features including poor eye contact, impairment in social interaction, impairment in communication, and repetitive and stereotypic behaviors. He also had a 5-year-old brother with developmental delay. Whole exome sequencing was performed on DNA isolated from peripheral blood samples of the patient and his parents. The sequence data were analyzed as a trio, and variants analysis was performed using ClinLabGeneticist software based on the following inheritance patterns: *de novo*, autosomal recessive and X-linked, and two *de novo* variants were identified.

The first *de novo* variant was identified in exon 32 of the *PCNXL2* gene, NM_014801.3: c.5626C>T, p.Arg1876Cys (hg19 Chr1:233134162). *PCNXL2* has not been described as a disease-related gene and there is limited information regarding its function. The variant is predicted to be damaging by SIFT and benign by PolyPhen-2. Sanger sequencing of DNA extracted from the patient, his parents and brother confirmed that the mutation occurred *de novo*.

The second *de novo* variant was identified in exon 6 of the *RPS2* gene, NM_002952.3: c.623C>T, p.Pro208Leu (hg19 Chr16:2012584). *RPS2* encodes a ribosomal protein that is a component of the 40S subunit. It has not been described as a disease-related gene and there is limited information regarding function, although recently it has been reported that *RPS2* is involved in dendritic spine maturation in rat hippocampal neurons [31]. The variant is predicted to be damaging by SIFT and benign by PolyPhen-2. Sanger sequencing of DNA extracted from the patient, his parents and brother confirmed that the mutation occurred *de novo*. Both *de novo* variant were interpreted to be of uncertain significance.

## Conclusions

Advancement of next generation sequencing technologies has provided an unprecedented opportunity in medicine, and we have entered a new era of genetic and genomic testing. However, a number of barriers need to be overcome before the full potential of WES in disease diagnosis and personalized medicine can be fully realized. A constant challenge in clinical genetic testing and molecular diagnosis is to interpret the clinical significance of variants with high confidence. It has been reported that some literature-annotated pathogenic variants are not truly ‘pathogenic’ [32, 33], and the issue is further manifested when large population exome data are examined [34]. Many variants in known disease genes that have been previously identified in specific disease cohorts occur at frequencies that are too high to support pathogenicity. Currently, there is no single comprehensive database with rigorously curated disease pathogenic variants. Therefore, it is critical to include all of the available variant annotation databases when genetic testing results are examined to assess their pathogenicity. Many commercially available variant analysis tools only include the most-commonly used population variant databases such as dbSNP and 1000 Genomes, or disease variant databases such as OMIM, HGMD, and ClinVar. ClinLabGeneticist incorporates to our knowledge, all publicly available variant databases, providing an extremely comprehensive genetic variant resource. Another issue in clinical genetic testing is the complexity of the process.

A unique feature of ClinLabGeneticist is that we implemented a logic table for variant interpretation at both gene level and variant level (Table 2). In the variant review process, it is first determined if the patient’s phenotype matches clinical features of the disease associated with the gene harboring the variant. Then pathogenicity of the variant is assessed. Decisions on variant validation and reporting are made based on both gene level and variant level assessments (Table 3). In contrast, currently available tools only allow variant level evaluation and these tools are more suitable for panel-based genetic testing where only known disease genes are tested. Clearly, ClinLabGeneticist is designed to enable a more comprehensive WES-based genetic testing. Another important feature of ClinLabGeneticist is it facilitates parallel variant review by multiple reviewers, including distributing variants to different reviewers, entry of variant analysis results by the reviewers, examining results by the administrators, and decision-making on final reporting. This complex process is managed more efficiently by ClinLabGeneticist than currently available tools.

In most clinical genetic laboratories, data management and process management efficiency is suboptimal, with many tasks handled manually. ClinLabGeneticist provides a platform to streamline and automate the workflow, not only significantly improving the efficiency and scalability, but also making the entire process less error-prone. We are currently generating WES data for an average of 30 trios per month and this scale can be readily handled by ClinLabGeneticist. We do not anticipate any technical issues if the number of WES-based testing increases to even several hundred trios per month. The challenge though is more reviewers are needed for variant assessment as the scale of WES goes up.

We also recognize the limitation of ClinLabGeneticist. Although patient clinical information is taken into consideration during variant assessment, it has not been incorporated into ClinLabGeneticist’s workflow. A new version of the software is being developed to improve on this aspect. In addition, currently ClinLabGeneticist is not amenable to analysis of disease associated copy number variation (CNV) or chromosome structural variation (SV). Therefore, although whole genome sequencing (WGS) platform is still supported by ClinLabGeneticist, only single nucleotide variants (SNVs) and small insertions/deletions would be analyzed. We will certainly revise the workflow and the tool when more clear guidelines on CNV and SV assessment become available.

## Availability and requirements

Project name: ClinLabGeneticist

Project home page: http://rongchenlab.org/software/clinlabgeneticist

Operating system(s): Windows

Programming language: Visual Basic, PHP, HTML

Other requirements: mySql

License: GNU, HGMD

Any restrictions to use by non-academics: please contact the authors

## Additional file

Additional file 1: Table S1. A sample variant input file for ClinLabGeneticist. **Table S2.** Variant annotation databases and features. **Table S3.** Summary statistic of variant review process for the three case studies. **Table S4.** Variant list for case 1. **Table S5.** Variant list for case 2. **Table S6.** Variant list for case 3. (XLSX 42 kb)
